# Complement activation in Hidradenitis suppurativa: Covert low-grade inflammation or innocent bystander?

**DOI:** 10.3389/fimmu.2022.953674

**Published:** 2022-09-21

**Authors:** K. R. van Straalen, K. Dudink, P. Aarts, H. H. van der Zee, T. P. P. van den Bosch, J. Giang, E. P. Prens, J. Damman

**Affiliations:** ^1^ Department of Dermatology, University of Michigan Medical School, Ann Arbor, MI, United States; ^2^ Department of Dermatology, Erasmus University Medical Center Rotterdam, Rotterdam, Netherlands; ^3^ Department of Pathology, Erasmus University Medical Center Rotterdam, Rotterdam, Netherlands; ^4^ Department of Pathology, Maasstad Hospital, Rotterdam, Netherlands; ^5^ Laboratory for Experimental Immunodermatology, Department of Dermatology, Erasmus University Medical Center Rotterdam, Rotterdam, Netherlands

**Keywords:** complement -innate immune system, C5a anaphylatoxin, treatment, immune system, bacteria, hidradenitis suppurativa

## Abstract

Hidradenitis suppurativa (HS) is a chronic auto-inflammatory skin disease with a complex and multifactorial pathogenesis involving both the innate and adaptive immune system. Despite limited evidence for *local* complement activation, conflicting results have been published on the role of *systemic* complement activation in HS. It was hypothesized that complement was consumed in highly inflamed HS skin, trapping complement from the circulation. Therefore, the aim of this study was to evaluate this *local* complement deposition in HS skin lesions using routine and commonly used complement antibodies.Direct immunofluorescence for C1q, C3c, C4d, C5b-9, and properdin was performed on frozen tissue sections of 19 HS patients and 6 controls. C5a receptor 1 (C5aR1) was visualized using immunohistochemistry.

Overall, we found no significant local complement deposition in HS patients versus controls regarding C1q, C3c, C4d, C5b-9, or properdin on either vessels or immune cells. C5aR1 expression was exclusively found on immune cells, predominantly neutrophilic granulocytes, but not significantly different relatively to the total infiltrate in HS lesions compared with controls. In conclusion, despite not being able to confirm local complement depositions of C1q, C3c, C4d, or properdin using highly sensitive and widely accepted techniques, the increased presence of C5aR1 positive immune cells in HS suggests the importance of complement in the pathogenesis of HS and supports emerging therapies targeting this pathway.

## Introduction

Hidradenitis suppurativa is a chronic auto-inflammatory skin disease with a complex and multifactorial pathogenesis (1). Both the innate and adaptive immune system are involved with upregulation of human beta-defensins, S100 proteins, complement components and a multitude of cytokines in lesional skin (2). Recent advances in HS treatment have seen the emergence of clinical trials with complement directed treatments such as vilobelimab (monoclonal antibody blocking C5a, InflaRx) and Avacopan (C5aR1 inhibitor, ChemoCentryx). However, the outcomes of these trials have been questioned due to the unclear primary outcome measure, HiSCR, that does not capture draining tunnels in a dynamic manner (3). Neither of these trials achieved its primary endpoint hampering the initial interpretation of the efficacy of these treatments in HS.

The rationale behind these clinical trials was based on the study by Kanni et al., who demonstrated systemic, late stage complement pathway activation in HS reflected by higher levels of C5a and C5b-9 in plasma of HS patients relative to controls (4). In contrast, our group found no increased circulating levels of C5a or sC5b-9 in patients with HS (5). Besides, concentrations of C3 and C3d as well as the C3d/C3 ratio were increased in patients with HS, but all, except for C3d, were within normal range. C3, C3b, iC3b and C4b were previously identified as downregulated differentially expressed proteins by Hoffman et al, who identified only C5a as an upregulated differentially expressed protein in the blood of HS patients (6). Remarkably, other inflammatory proteins with a known correlation to systemic complement activation such as IL-6 and CRP have not been found consistently elevated in plasma of HS patients (4, 5).

Besides systemic complement activation, several studies identified increased expression of complement genes using different RNA sequencing methods (7–9). Hotz et al. found increased RNA expression of C1q, C2, CR1, C3aR1, C5aR1, and Factor B and decreased expression of C7, Factor H, and Factor D in HS lesional skin (8). More recently, Gudjonsson et al. showed complement activation among the significantly enriched biological processes in both bulk RNA seq of HS lesional skin and plasma cells derived from single cell RNA sequencing of lesional skin (9). Moreover, this study demonstrated increased protein levels of C1q, C3b, C4d and complement receptors CR1 and CR2 using immunohistochemistry on formalin fixed paraffin embedded (FFPE) lesional slides of three HS patients (9).

**Figure 1:**
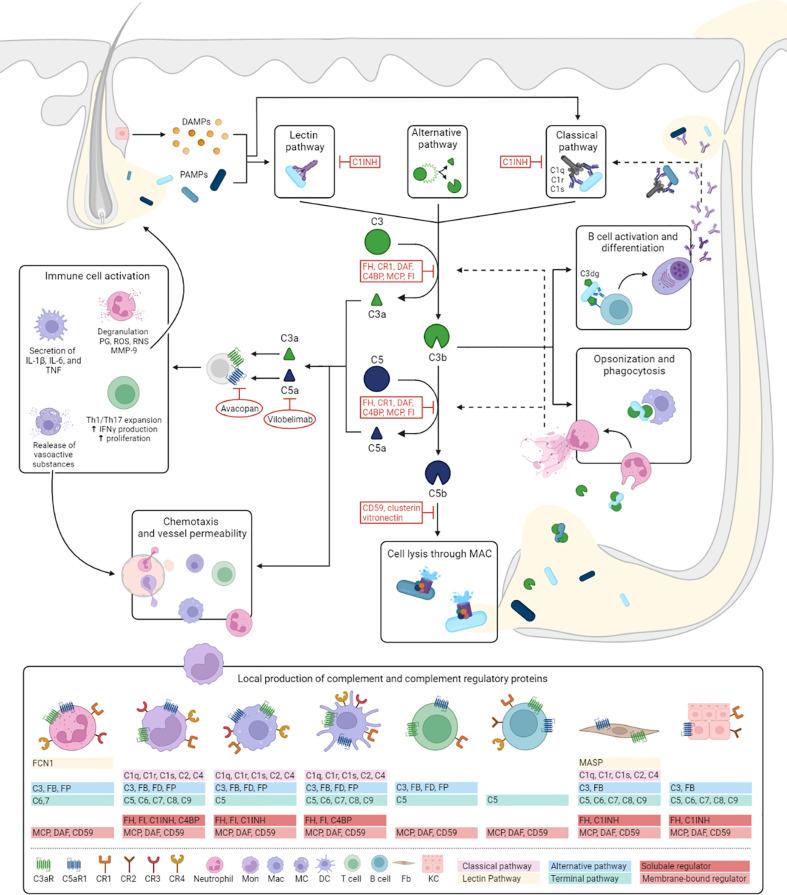
Hypothesized role of complement in HS. Despite finding no evidence for the activation of complement in our study, complement proteins have several well-known functions which are likely to contribute to HS pathogenesis. This figure illustrates the mechanisms of activation and regulation of the complement cascade and outlines how it’s effector functions could fit into the pathogenesis of HS. The complement cascade can be activated by three different pathways: the classical, lectin and alternative pathway. The classical pathway is triggered by antibody-antigen complexes, cell particles and acute phase proteins binding to C1 (consisting of C1q, C1r, and C1s), and is therefore more likely to play a more prominent role in later stages of the diseases. Activation of the lectin pathway occurs through the binding of the complex of mannose-binding lectin or ficolins to PAMPs or DAMPs released from dilated and ruptured follicles and tunnels. Both pathways result in the activation of C4 and C2, to form the C3 convertase, C4b2a. The alternative pathway depends on constitutive spontaneous low-grade hydrolysis of C3, ultimately forming the alternative C3-convertase C3bBb. All pathways converge on the cleavage of C3 into C3a and C3b. C3b forms a C5 convertase by combining with previous C3 convertases initiating the terminal complement pathway by cleaving of C5 into C5a and C5b. C5b forms a complex with C6 and C7, which is inserted in the cell membrane. Subsequently, C8 and multiple C9 molecules bind to this complex, resulting in a fully functional MAC (C5b-9) capable of lysing for example bacteria expelled from ruptured follicles and tunnels or direct attack on epithelial cells. C3a and C5a are potent anaphylatoxins resulting in increased vessel permeability and further attracting monocytes/macrophages, neutrophils, mast cells and T cells (C5a) to the HS lesion. These cells are also capable of locally producing complement components. In addition, interaction of C3a with C3aR and C5a with C5aR1 induces macrophages to secrete HS associated pro-inflammatory cytokines, increases proliferation of T cells, and promotes Th1 and Th17 expansion, cell types that play prominent role in HS pathogenesis. In addition, regulatory T-cell generation and function are decreased by C3a and C5a (not shown in figure). Activation of neutrophils leads to the release of prostaglandins, reactive oxygen and reactive nitrogen species, and MMP-9. The latter potentially aiding the characteristic HS tissue destruction. Two therapies, vilobelimab and avacopan, aimed neutralizing C5a and blocking C5aR1 respectively, have shown promising preliminary results in HS. Bacterial opsonization with (i)C3b facilitates phagocytosis by macrophages and neutrophils and has been shown to stimulate NETosis. Prominent NET formation found in chronic HS lesions could be contributing to further complement activation through for example MPO and cathepsin G depositions on extruded NETs which are able to respectively cleave C3 and C5. Recognition of C3dg-coated bacteria by antigen-specific B-cell receptors and CR2 lower the threshold of B cell activation and promotes antibody production. Local secretion of antibodies may in turn aid activation of the classical pathway. C1INH, C1 esterase inhibitor; CR, complement receptor; DAF, decay-accelerating factor; DAMPs, danger associated molecular patterns; DC, dendritic cell; FB, Factor B; Fb, fibroblast; FCN1, ficolin 1; FD, Factor D; FH, Factor H; FI, Factor I; FP, Factor P; MAC, membrane attack complex; Mac, macrophage; MC, mast cell; MASP, mannose-associated serine protease; MCP, Membrane cofactor protein; NET, neutrophil extracellular trap; PAMPs, pathogen associated molecular patterns; PG, prostaglandins; ROS, reactive oxygen species; RNS, reactive nitrogen species.

To date, no pathogenic variants in complement genes have been found in HS. However, sufficiently large genome wide association studies have not yet been performed in HS. These studies might yield polymorphisms associated with complement genes as these have been found in HS-associated diseases such as psoriasis. Nonetheless, based on the pathogenesis of HS, activation of the complement pathway seems indisputable and is hypothesized to play a multifactorial role in the disease process (**Figure 1**). However, due to the conflicting results of the two published studies on systemic complement activation, it was hypothesized that complement might be consumed in highly inflamed HS skin, locally trapping complement from the circulation (4, 5). To support this theory, we aimed to provide high quality evidence of local complement activation in HS lesional skin by visualizing complement components using direct immunofluorescence (DIF) on frozen sections in accordance with routine diagnostic procedures for diseases such as auto-immune bullous diseases and IgA-vasculitis, using widely accepted specific complement antibodies.

## Materials and methods

### Patient selection

Lesional skin was obtained from HS patients who underwent routine excisional surgery in the Erasmus Medical Center Rotterdam, Rotterdam, the Netherlands. Skin considered surgical discard after mamma reduction surgery was used as control. Under the prevailing opt-out principle and ethical guidelines in the Erasmus MC, excised skin is considered surgical discard and exempt from informed consent or ethical approval. Patient baseline characteristics for HS patients were obtained from electronic patient files.

### Sample preparation

Skin excision specimens were cut manually using the bread-loaf technique in which each specimen was cut transversally at 4 mm intervals. One central segment was frozen at -80 degrees and stored for DIF. The remaining segments were FFPE embedded, tissue sections from these segments were stained with hematoxylin and eosin (H&E) (or immunohistochemical staining) for light microscopic evaluation.

### Direct immunofluorescence

Direct immunofluorescence was performed on the stored frozen material as previously described by Damman et al. (10). Additionally, frozen sections were stained with H&E for light microscopic evaluation. For detection of complement factors, DIF was performed by automated immunofluorescent staining using the Ventana Benchmark Discovery (Ventana Medical Systems Inc.). Wet slides were loaded and incubated with the primary antibody of interest for 32 min at 37 ^0^C followed by detection with either omnimap-anti rabbit or mouse, labeled with HRP and visualized with FAM (#760–243, Ventana). Slides were covered with anti-fading medium (DAKO, S3023). DIF intensity and area of vascular and immune infiltrate was scored by two independent pathologists (J.G. and J.D.). The DIF intensity was scored on a nominal scale of 0–3: none (0), weak (1), moderate (2), profound/bright (3). As the number of immune cells and vessels were found to differ between Hurley stages, the percentage positive vessels and immune infiltrate relative to the total number of vessels or immune cells was scored on a nominal scale: none (0), < 5% of positive (1), 5-50% positive (2), and > 50% positive (3). A cumulative score was calculated by multiplying the intensity x area. Markers that were included for analysis were C1q, C4d, properdin, C3c and C5b-9, covering classical (C1q, C4d), alternative (properdin), lectin and classical (C4d) and terminal pathway activation (C5b-9). See **Supplemental Table 1** for antibodies used.

### C5aR1 immunohistochemistry

Immunohistochemistry was performed for C5aR1 using a primary monoclonal antibody to human C5aR1, clone S5/1. Detection for C5aR1 was identical as described for DIF, except for the use of Diaminobenzidine (DAB) as detection substrate. The percentage positive immune cells relative to the total of immune cells was scored on a nominal scale: none (0), < 5% positive (1), 5-50% positive (2), and > 50% positive (3). This scoring was performed for the whole slide and per tunnel (when present) separately. For the infiltrate, a distinction was made between granulocyte or histiocyte positive C5aR1 cells, based on morphology.

### Statistics

Differences in the nominally scored intensity of staining and the overall cumulative score for staining between HS patients and healthy controls were assessed using Chi-square tests. These were performed for both complement factors in blood vessels and on polymorphonuclear leukocytes (PMNs). Comparisons were performed for the overall HS group versus healthy controls as well as for each individual Hurley stage compared with controls and the other Hurley stages. Additionally, for C5aR1 analyses have been performed comparing the stained cell types as well as the effect of tunnels, stratified for different Hurley stages. Statistical analyses were performed using SPSS (IBM Corp. Released 2016. IBM SPSS Statistics for Windows, Version 28.0.1.0 Armonk, NY.) and two-sided p-values ≤0.05 were considered statistically significant.

## Results

### Patient characteristics

In total, excisional samples from 19 HS patients and 6 controls were included. The majority of included HS samples were obtained from male patients (58%), all control samples were obtained from women. All Hurley stages were represented with 32% of samples obtained from patients with Hurley stage I, 42% from Hurley stage II patients, and 26% from Hurley stage III patients (**Table 1**). While all skin from healthy donors was derived from the mammae, HS samples were derived from the buttocks (47%), axillae (26%), groin (16%), or abdomen (11%).

**Table 1:** Patient characteristics.

	Total n = 19		Hurley I n = 6		Hurley II n = 8		Hurley II n = 5	
**Sex, *n* (%)**								
Female	8	(42)	3	(50)	4	(50)	1	(20)
**Age**, median [IQR]*	45.3	[32.0-55.2]	46.7	[31.8-50.8]	48.8	[38.0-56.7]	32.0	[20.8-56.4]
**Age of onset**, median [IQR]*	26.8	[15.5-36.0]	30.0	[20.3-39.8]	20.0	[13.5-12.4]	29.0	[17.0-38.5]
**Disease duration years**, median [IQR**]**	14.7	[3.6-23.0]	12.8	[4.3-17.8]	20.3	[12.4-32.8]	3.1	[3.0-18.6]
**BMI**, median [IQR]**	27.3	[24.5-30.6]	26.3	[23.6-30.8]	25.3	[23.9-30.5]	26.0	[25.1-29.4]
**Smoking status**, *n* (%)								
Current or ex-smoker	14	(74)	5	(83)	6	(75)	3	(60)
**Location** *, n* (%)								
Axillae	5	(26)	1	(17)	2	(25)	2	(40)
Groin	3	(16)	2	(33)	1	(13)	0	(0)
Buttocks	9	(47)	3	(50)	3	(38)	3	(60)
Abdomen	2	(11)	0	(0)	2	(25)	0	(0)

IQR, Interquartile range; BMI, Body mass index. ^*^ Missing data for 2 patients ^**^ Missing data for 1 patient.

### Immune infiltrate in complement-stained tissue sections

In general, sections from Hurley stage I were characterized by moderate to severe deep dermal and subcutaneous active inflammation with influx of many neutrophils, sometimes with abscess formation and admixture of eosinophils. Hurley I lesions frequently showed granulomatous foreign body reaction on ruptured hair follicles. A few Hurley stage I lesions showed dermally located epithelial strands, potentially as remnants from a ruptured abscess, and dermal scarring. Hurley stage II sections exhibited moderate chronic lymphoplasmacellular and active inflammation, frequently associated with tunnel formation, moderate foreign body reaction and moderate scarring. Sections obtained from Hurley stage 3 lesions showed profound chronic lymphoplasmacellular inflammation, mild to moderate active inflammation, mild and focal granulomatous inflammation, and profound scarring.

### Local complement deposition in HS lesions

**Figure 2:**
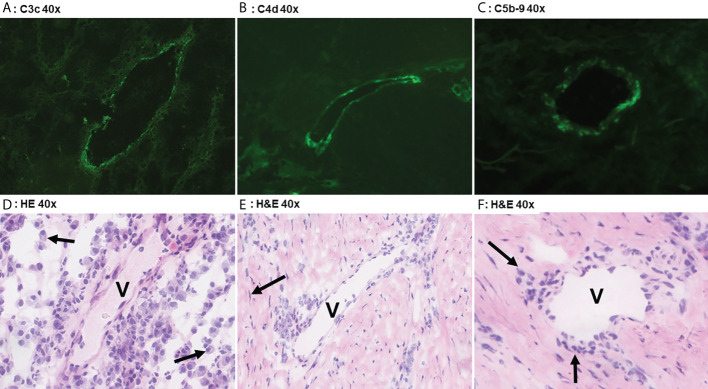
Direct immunofluoresence of complement components. Direct immunofluorescence findings in a case of Hurley stage 3 (panel **A**) shows focal moderate vascular endothelial C3c staining in a single vessel (V). Panel **D** shows the corresponding frozen H&E section with profound plasmacellular perivascular inflammation (solid arrows). Panel **B** shows strong focal vascular endothelial staining for C4d in a single vessel in Hurley stage 3 and the corresponding H&E **(E)** demonstrates mild-to-moderate lymphohistiocytic perivascular inflammation. Note the surrounding fibrosis with scattered fibroblast (solid arrow) in parallel distribution. Weak C5b-9 deposition in a vessel of Hurley stage 2 (Panel **C**) in which the H&E **(F)** reveals moderate chronic inflammation with perivascular lymphohistiocytic inflammation (solid arrows). **(A)** C3c 40x. **(B)** C4d 40x. **(C)** C5b-9 40x. **(D)** H&E 40x. **(E)** H&E 40x. **(F)** H&E 40x.

C3c could only be detected in vessels of 3/19 HS patients (16%) with a moderate intensity in <5% of the total number of vessels, **Table 2**. Positive staining for C3c was only found in patients with Hurley stages 2 or 3, while C3c staining was absent in all Hurley stage I patients and all healthy controls (**Table 3**). Vascular C4d was found moderately to strongly positive in 12/19 HS (63%, Hurley I-III) in predominantly <5% of the total numbers of vessels. However, similar findings were also observed in 2/6 (33%) healthy controls. Vascular C5b-9 could be demonstrated in 5/19 of HS patients (26%), all of which were Hurley stage II or III, but also only in <5% of the total numbers of vessels present. C1q, a marker of early classical complement pathway activation, was detected in only 1/19 HS patients (Hurley stage III) in <5% of vessels, **Tables 2**, **3**. Vascular properdin was not detected in any of the HS or control samples. **Figure 2** shows representative DIF staining of the focal vascular depositions of C3c (Hurley stage III), C4d (Hurley stage III) and C5b-9 (Hurley stage II) in HS patients.

**Table 2:** Direct immunofluorescence intensity and cumulative scores in HS versus healthy controls for blood vessels.

	Hidradenitis	Suppurativa (n = 19)	Healthy	Controls (n = 6)	P-value	
	Intensity*	cumulative^+^	intensity*	cumulative^+^	intensity	cumulative
**C3c**	0 (84)	0 (84)	0 (100)	0 (100)	0.584	0.584
	1 (5)	1 (5)	1 (0)	1 (0)		
	2 (11)	2 (11)	2 (0)	2 (0)		
	3 (0)	3 (0)	3 (0)	3 (0)		
**C4d**	0 (47)	0 (47)	0 (67)	1 (67)	0.114	0.132
	1 (0)	1 (0)	1 (0)	2 (0)		
	2 (42)	2 (42)	2 (0)	3 (0)		
	3 (11)	3 (5)	3 (33)	6 (17)		
		9 (5)		9 (17)		
**C5b-9**	0 (74)	0 (74)	0 (100)	0 (100)	0.373	0.373
	1 (10)	1 (5)	1 (0)	1 (0)		
	2 (16)	2 (21)	2 (0)	2 (0)		
	3 (0)	3 (0)	3 (0)	3 (0)		
**Properdin**	0 (100)	0 (100)	0 (100)	0 (100)	n.c.	n.c.
	1 (0)	1 (0)	1 (0)	1 (0)		
	2 (0)	2 (0)	2 (0)	2 (0)		
	3 (0)	3 (0)	3 (0)	3 (0)		
**C1q**	0 (95)	0 (95)	0 (100)	0 (100)	0.566	0.566
	1 (5)	1 (5)	1 (0)	1 (0)		
	2 (0)	2 (0)	2 (0)	2 (0)		
	3 (0)	3 (0)	3 (0)	3 (0)		

All data is expressed as scoring category (%). PMN, Polymorphonuclear leukocyte; n.c, not calculated. *Intensity was scored on a nominal scale of 0–3: none (0), weak (1), moderate (2), profound/bright (3). ^+^Cumulative score was calculated by multiplying the intensity x area.

**Table 3:** Subgroup analysis of direct immunofluorescence intensity and cumulative scores for different Hurley stages in HS for blood vessels.

	Hurley I		Hurley II		Hurley III		P-value	
	intensity*	Cumulative^+^	intensity*	Cumulative^+^	intensity*	Cumulative^+^	intensity	Cumulative
**C3c**	0 (100)	0 (100)	0 (88)	0 (88)	0 (60)	0 (60)	0.352	0.352
	1 (0)	1 (0)	1 (0)	1 (0)	1 (20)	1 (20)		
	2 (0)	2 (0)	2 (13)	2 (13)	2 (20)	2 (20)		
	3 (0)	3 (0)	3 (0)	3 (0)	3 (0)	3 (0)		
**C4d**	0 (50)	0 (50)	0 (75)	0 (75)	0 (0)	0 (0)	0.111	0.088
	1 (0)	1 (0)	1 (0)	1 (0)	1 (0)	1 (0)		
	2 (33)	2 (33)	2 (25)	2 (25)	2 (80)	2 (80)		
	3 (17)	3 (17)	3 (0)	3 (0)	3 (20)	9 (20)		
**C5b-9**	0 (63)	0 (63)	0 (63)	0 (63)	0 (60)	0 (60)	0.501	0.357
	1 (12)	1 (12)	1 (13)	1 (13)	1 (20)	1 (20)		
	2 (25)	2 (25)	2 (25)	2 (25)	2 (20)	2 (20)		
	3 (0)	3 (0)	3 (0)	3 (0)	0 (0)	0 (0)		
**Properdin**	0 (100)	0 (100)	0 (100)	0 (100)	0 (100)	0 (100)	n.c.	n.c.
	1 (0)	1 (0)	1 (0)	1 (0)	1 (0)	1 (0)		
	2 (0)	2 (0)	2 (0)	2 (0)	2 (0)	2 (0)		
	3 (0)	3 (0)	3 (0)	3 (0)	3 (0)	3 (0)		
**C1q**	0 (100)	0 (100)	0 (100)	0 (100)	0 (80)	0 (80)	0.228	0.228
	1 (0)	1 (0)	1 (0)	1 (0)	1 (20)	1 (20)		
	2 (0)	2 (0)	2 (0)	2 (0)	2 (0)	2 (0)		
	3 (0)	3 (0)	3 (0)	3 (0)	3 (0)	3 (0)		

All data is expressed as scoring category (%).n.c, not calculated. *Intensity was scored on a nominal scale of 0–3: none (0), weak (1), moderate (2), profound/bright (3). ^+^Cumulative score was calculated by multiplying the intensity x area.

Immune cells throughout all HS sections were found to be negative for any of the complement components stained through DIF, regardless of disease stage.

Overall, statistical analyses revealed that there were no significant differences between either the intensity or cumulative scores for any C1q, C3c, C4d, or properdin in HS slides compared with healthy controls.

**Figure 3:**
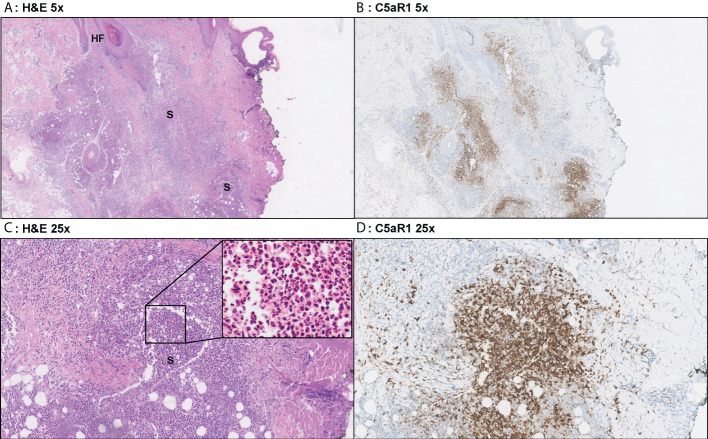
Representative C5aR1 staining in Hurley stage I patient. Panel **A** (5x magnification) and **C** (25x) show representative H&E sections of a Hurley stage I patient with extensive deep dermal and subcutaneous suppurative (S) abscessing inflammation surrounding hair follicles with hyperkeratosis (HF). The inset in panel C shows that the infiltrate almost exclusively exists of neutrophils. Panels **B**, **D** shows that a large part of these neutrophils are C5aR1 positive. **(A)** H&E 5x. **(B)** C5aR1 25x. **(C)** H&E 5x. **(D)** C5aR1 25x.

**Figure 4:**
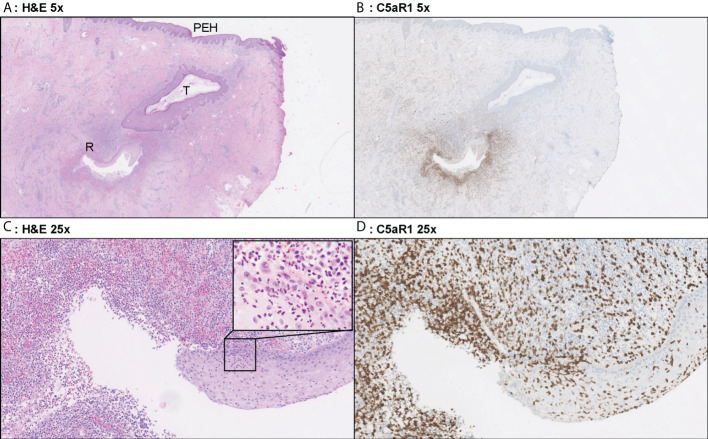
Representative C5aR1 staining in Hurley stage II patient. Panel **A** (5x magnification) and **C** (25x) show representative H&E sections of a Hurley stage II patient with tunnel formation (T) in which the tunnels are covered with hyperplastic squamous cells epithelium. Note the psoriasiform epidermal hyperplasia (PEH), characteristic for late-stage HS lesions. Deeper in the excision tunnel rupture (R) (occurs and the epithelium is surrounded by sheets of neutrophils. The inset in panel **C** (40x) shows the profound transepithelial migration of neutrophils. Panels **B**, **D** shows that a large part of these neutrophils are C5aR1 positive. **(A)** H&E 2.5x. **(B)** C5aR1 2.5x. **(C)** H&E 25x. **(D)** C5aR1 25x.

**Figure 5:**
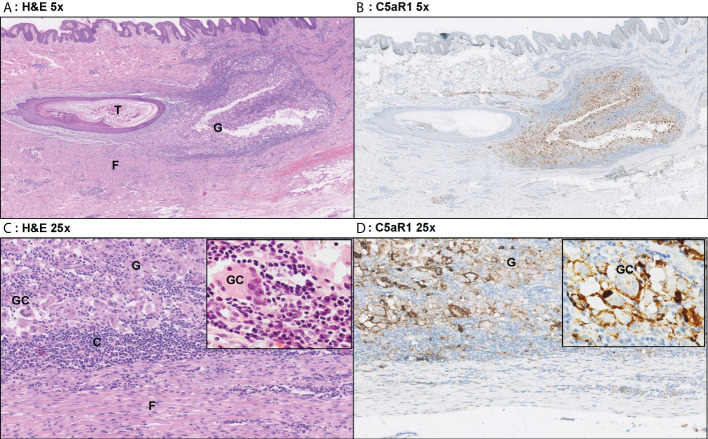
Representative C5aR1 staining in Hurley stage III patient. Panel **A** (5x magnification) and **C** (25x) show representative H&E sections of a Hurley stage III patient with tunnel formation (T) with luminal hyperkeratosis and profound surrounding granulomatous (G) inflammation with foreign body giant cells (GC) and extensive fibrosis/scarring (F). The inset (40x) shows a foreign body giant cell surrounded by many plasma cells. Panels **B**, **D** show the corresponding C5aR1 stainings in which many histiocytes and giant cells are C5aR1 positive. Inset 40x. **(A)** H&E 5x. **(B)** C5aR1 5x. **(C)** H&E 25x. **(D)** C5aR1 25x.

**Figure 6:**
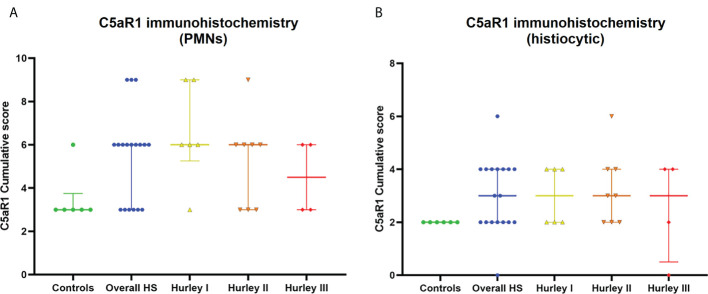
C5aR1 staining of different immune cell types. Cumulative score of C5aR1 staining for the control group plotted separately for PMNs **(A)** and histiocytes **(B)**, overall HS group and per Hurley category. PMN, polymorphonuclear leukocyte.

**Figure 7:**
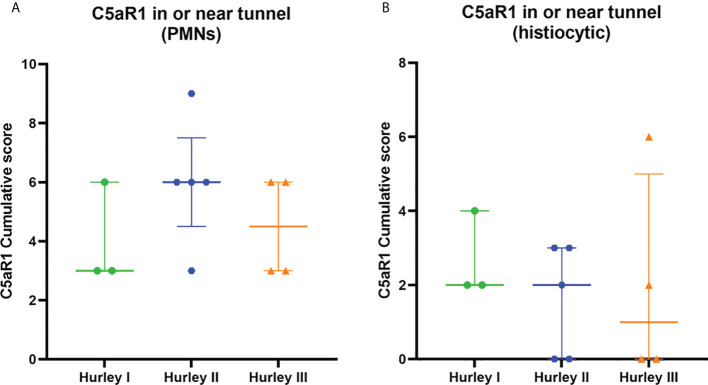
C5aR1 staining in relation to dermal tunnels. Cumulative score of C5aR1 staining in or near tunnels stratified per Hurley category, plotted separately for PMNs **(A)** and histiocytes **(B)**. Association of C5aR1 positive immune cells with epithelial remnants of ruptured abscesses were scored for Hurley (I) PMN, polymorphonuclear leukocyte.

In contrast, C5aR1 expression was exclusively found on immune cells. Representative staining of C5aR1 in different Hurley stages can be found in **Figures 3**–**5**. C5aR1 expression was predominantly found on granulocytes and to a lesser extent on histiocytes and giant cells (**Figure 6**). A marked increase in C5aR1^+^ PMNs was seen in HS samples, particularly Hurley stage I samples, compared with controls. However, as staining was scored relative to the infiltrate size the difference was not statistically significant for any of the Hurley stages versus controls or between Hurley stages. There was no increased number of C5aR1^+^ immune cells found in sections with tunnels compared to sections without tunnels (**Figure 7**). Accompanying the localization of neutrophils, C5aR1 staining was most pronounced directly adjacent to ruptured cysts or dermal tunnels.

## Discussion

In recent years, conflicting results have been published on the role of *systemic* complement activation in HS. Although our group could not find activation of circulating complement components, others previously demonstrated significantly increased levels of C5a and C5b-9 in the circulation of HS patients (4). To explain this difference, it was hypothesized that complement might be consumed in highly inflamed HS skin, trapping complement from the circulation. The aim of this study was to evaluate this local complement deposition in the skin in frozen HS skin lesions using routine and widely used complement antibodies. Surprisingly, but in line with our previously published findings in plasma, we could not demonstrate significant local complement deposition in HS versus controls. Although we found focal endothelial C3c, C4d, and C5b-9 deposition, this was not significantly different from controls. Moreover, one could even argue the clinical relevance of these focal findings: it is well known that vascular complement deposition (and immunoglobulins) can be found in several types of dermatitis and even in normal skin, all in the absence of vasculitis. The finding of an almost lack of complement deposition in our study is in contrast to previous findings: using immunohistochemistry on three patients, Gudjonsson and colleagues demonstrated increased complement deposition on immune cells, particularly in the deeper layers of the dermis (9). The discrepancy could be explained by several factors: Firstly, there is a difference in tissue used, where we used frozen material, Gudjonsson et al. used FFPE. Complement staining tends to have the highest sensitivity and specificity on frozen material. Secondly, different antibodies were used, we worked with commonly accepted antibodies that are widely used in routine diagnostics and in the field of complement research. Thirdly, the life of lesions biopsied could influence results: comparable to immunoglobulins, complement factors have a certain tissue residency and complement depositions could be missed in older lesions as they are actively cleared. Lastly, low-grade complement activation in HS might be highly locally regulated by membrane bound complement regulator proteins such as soluble complement receptor 1 (SCR1/CD35), decay accelerating factor (DAF, CD55) and CD59.

It is worth mentioning that we found linear C4d, properdin and C5b-9 basement membrane (BM) deposits as well as focal arteriolar deposits of C3c, C4d and C5b-9. These findings parallel observations in the kidney in which arteriolar C5b-9 staining are considered an internal positive control and are of non-specific significance (11, 12). Based on several studies in the kidney it is hypothesized that small-to medium sized vascular and (tubular) BM staining for C3, C4 and C5b-9 in healthy subjects might be explained by localized cellular injury acquired during aging, as a consequence of tissue sampling, or on the epidermal BM as proteins involved in the first line of defense against pathogens (12, 13).

Although we did not find significant local cutaneous complement deposition, our findings however, do not exclude a role for complement in HS pathogenesis. Hypothetically, clear parallels could be drawn between the involvement of complement in HS and in granulomatosis with polyangiitis (GPA). In the latter, scarce immunoglobulin deposits in lesional biopsies (therefore labelled ‘pauci‐immune’) were originally considered as evidence for negligible complement activation (14). During the last decade however, up to one-third of patients were found to show complement deposition in their renal biopsies (15). The relevance of complement activation in GPA is illustrated by the fact that Avacopan has been registered for the treatment of GPA and shows clear beneficial results (16). Although there is a different pathogenesis underlying these diseases, a comparable adverse role of C5a-C5aR1-axis activation in GPA might also play a role in HS, despite almost negative DIF results. This is supported by preliminary results from a phase 2 randomized controlled trial in HS that demonstrated effectivity of Avacopan (30 mg twice-daily) in patients with Hurley stage III in secondary outcome measures; IHS4 and the abscess and inflammatory nodule (AN) count (17). Local activation of complement is further supported by our data showing expression of C5aR1 on multiple immune cells in HS lesional skin. Similarly, the vilobelimab (IFX‐1, a monoclonal antibody that selectively binds to C5a) demonstrated a significantly greater reduction in the number of draining tunnels in the highest dosed treatment group relative to the placebo group at week 16 (18).

Interestingly, both Avacopan and vilobelimab were shown to be more efficacious in patients with severe disease (Hurley stage III) and/or tunnels (17, 18). This is likely due to a difference in the contribution of complement in different disease stages. Acute lesions due to rupture of hair follicles resolve spontaneously, indicating that activation of the complement pathway in these lesions is likely adequately regulated by membrane bound regulator proteins and short lived. In contrast, severe, chronic lesions are characterized by non-resolving inflammation arising from partially epithelialized or ruptured tunnels. These lesions show active but predominantly chronic lymphoplasmacellar inflammation, prominent NETosis and decreased NET degradation, **Figure 1** (9, 19). In these lesions, the large amount of locally secreted immunoglobulins (incl. autoantibodies) combined with the ongoing exposure to bacteria is likely a continuous driver of complement activation. It is therefore counterintuitive that we did not find an increased abundance of C5aR1 positive immune cells in slides with tunnels compared to those without. Additionally, proteins expressed by activated neutrophils during degranulation and excreted on NETs further attract and activate neutrophils in a potentially vicious cycle (20, 21). Inhibiting this vicious cycle of complement activation in severe disease by administration of Avacopan or vilobelimab is therefore likely to show greater clinical effect then inhibiting an already adequately regulated complement response in milder patients (17, 18). A large infiltrate in both mild and severe disease, however, could not only lead to complement activation it could also contribute to quick degradation of (otherwise long lived) complement components, potentially making them relatively difficult to detect on the protein level.

A novel finding was the association of C5aR1 staining on both histiocytes and giant cells. Loosely structured granulomatous inflammation and foreign body giant cells containing keratin fragments have previously been described in HS lesions (1, 22). However, this granulomatous inflammation likely also represents an attempt to limit the bacterial loads from ruptured or non-epithelialized tunnels. While the exact contribution of C5a to this process in HS remains unknown, data from other diseases indicate that C5a plays an important role in the mediation of chemotaxis and activation events required for the formation of a granulomatous response (23, 24).

In conclusion, despite not being able to confirm local complement depositions of C1q, C3c, C4d, properdin, or C5b-9 using highly sensitive and widely accepted techniques, the increased presence of C5aR1 on immune cells in HS indicates the importance of complement in the pathogenesis of HS and provide support for the emerging therapies targeting this pathway. Future studies assessing other complement receptors and membrane bound complement regulator proteins could shed more light on the activation of the complement pathway in HS lesional skin.

## Data availability statement

The original contributions presented in the study are included in the article/**Supplementary Material**. Further inquiries can be directed to the corresponding author.

## Ethics statement

Ethical review and approval was not required for the study on human participants in accordance with the local legislation and institutional requirements. Written informed consent for participation was not required for this study in accordance with the national legislation and the institutional requirements.

## Author contributions

Conceptualization: EP, JD; Methodology: JD, TB; Formal analysis: JD, JG, TB, KD; Resources: EP, JD; Data Curation: HZ, EP; Writing - Original Draft: KS, JD; Writing - Review & Editing: KS, KD, PA, HZ, TB, JG, EP, JD; Visualization: KD, JD; Supervision: EP, JD. All authors contributed to the article and approved the submitted version.

## Conflict of interest

The authors declare that the research was conducted in the absence of any commercial or financial relationships that could be construed as a potential conflict of interest.

## Publisher’s note

All claims expressed in this article are solely those of the authors and do not necessarily represent those of their affiliated organizations, or those of the publisher, the editors and the reviewers. Any product that may be evaluated in this article, or claim that may be made by its manufacturer, is not guaranteed or endorsed by the publisher.

## References

[1] van Straalen KR Prens EP Gudjonsson JE. Insights into hidradenitis suppurativa. J Allergy Clin Immunol (2022) 149(4):1150–61. doi: 10.1016/j.jaci.2022.02.003 35189127

[2] Vossen A van der Zee HH Prens EP. Hidradenitis suppurativa: A systematic review integrating inflammatory pathways into a cohesive pathogenic model. Front Immunol (2018) 9:2965. doi: 10.3389/fimmu.2018.02965 30619323 PMC6302105

[3] van Straalen KR Ingram JR Augustin M Zouboulis CC. New treatments and new assessment instruments for hidradenitis suppurativa. Exp Dermatol (2022). doi: 10.1111/exd.14609 PMC9542859 35582833

[4] Kanni T Zenker O Habel M Riedemann N Giamarellos-Bourboulis EJ. Complement activation in hidradenitis suppurativa: A new pathway of pathogenesis? Br J Dermatol (2018) 179(2):413–9. doi: 10.1111/bjd.16428 29405257

[5] Prens LM Ardon CB van Straalen KR van der Zee HH Seelen MAJ Laman JD. No evident systemic terminal complement pathway activation in hidradenitis suppurativa. J Invest Dermatol (2021) 141(12):2966–9 e1. doi: 10.1016/j.jid.2021.03.037 34252397

[6] Hoffman LK Tomalin LE Schultz G Howell MD Anandasabapathy N Alavi A. Integrating the skin and blood transcriptomes and serum proteome in hidradenitis suppurativa reveals complement dysregulation and a plasma cell signature. PloS One (2018) 13(9):e0203672. doi: 10.1371/journal.pone.0203672 30265680 PMC6162087

[7] Blok JL Li K Brodmerkel C Jonkman MF Horváth B. Gene expression profiling of skin and blood in hidradenitis suppurativa. Br J Dermatol (2016) 174(6):1392–4. doi: 10.1111/bjd.14371 26707687

[8] Hotz C Boniotto M Guguin A Surenaud M Jean-Louis F Tisserand P. Intrinsic defect in keratinocyte function leads to inflammation in hidradenitis suppurativa. J Invest Dermatol (2016) 136(9):1768–80. doi: 10.1016/j.jid.2016.04.036 27206704

[9] Gudjonsson JE Tsoi LC Ma F Billi AC van Straalen KR Vossen ARJV. Contribution of plasma cells and B cells to hidradenitis suppurativa pathogenesis. JCI Insight (2020) 5(19):e139930. doi: 10.1172/jci.insight.139930 PMC7566715 32853177

[10] Damman J Mooyaart AL Bosch T Seelen MA Doorn MBV. Lectin and alternative complement pathway activation in cutaneous manifestations of iga-vasculitis: A new target for therapy? Mol Immunol (2022) 143:114–21. doi: 10.1016/j.molimm.2022.01.011 35121432

[11] Dumont C Mérouani A Ducruet T Benoit G Clermont MJ Lapeyraque AL. Clinical relevance of membrane attack complex deposition in children with iga nephropathy and henoch-schönlein purpura. Pediatr Nephrol (2020) 35(5):843–50. doi: 10.1007/s00467-019-04445-x 31932958

[12] Koopman JJE van Essen MF Rennke HG de Vries APJ van Kooten C. Deposition of the membrane attack complex in healthy and diseased human kidneys. Front Immunol (2020) 11:599974. doi: 10.3389/fimmu.2020.599974 33643288 PMC7906018

[13] Basset-Seguin N Dersookian M Cehrs K Yancey KB. C3d,G is present in normal human epidermal basement membrane. J Immunol (1988) 141(4):1273–80. 3135326

[14] Jennette JC Wilkman AS Falk RJ. Anti-neutrophil cytoplasmic autoantibody-associated glomerulonephritis and vasculitis. Am J Pathol (1989) 135(5):921–30. PMC1880110 2683800

[15] Chen M Daha MR Kallenberg CG. The complement system in systemic autoimmune disease. J Autoimmun (2010) 34(3):J276–86. doi: 10.1016/j.jaut.2009.11.014 20005073

[16] Jayne DRW Merkel PA Schall TJ Bekker P Group AS. Avacopan for the treatment of anca-associated vasculitis. N Engl J Med (2021) 384(7):599–609. doi: 10.1056/NEJMoa2023386 33596356

[17] ChemoCentryx. Chemocentryx announces positive topline results of phase ii aurora clinical trial of avacopan in the treatment of hidradenitis suppurativa (HS). (2020).

[18] inflaRx. Inflarx reports additional analysis of the shine phase lib results for ifx-1 in hidradenitis suppurativa. (2019).

[19] Byrd AS Carmona-Rivera C O'Neil LJ Carlucci PM Cisar C Rosenberg AZ. Neutrophil extracellular traps, B cells, and type I interferons contribute to immune dysregulation in hidradenitis suppurativa. Sci Transl Med (2019) 11(508):eaav5908. doi: 10.1126/scitranslmed.aav5908 31484788 PMC11369904

[20] Carmona-Rivera C O'Neil LJ Patino-Martinez E Shipman WD Zhu C Li QZ. Autoantibodies present in hidradenitis suppurativa correlate with disease severity and promote the release of proinflammatory cytokines in macrophages. J Invest Dermatol (2022) 142(3 Pt B):924–35. doi: 10.1016/j.jid.2021.07.187 PMC8860851 34606886

[21] de Bont CM Boelens WC Pruijn GJM. Netosis, complement, and coagulation: A triangular relationship. Cell Mol Immunol (2019) 16(1):19–27. doi: 10.1038/s41423-018-0024-0 29572545 PMC6318284

[22] van der Zee HH de Ruiter L Boer J van den Broecke DG den Hollander JC Laman JD. Alterations in leucocyte subsets and histomorphology in normal-appearing perilesional skin and early and chronic hidradenitis suppurativa lesions. Br J Dermatol (2012) 166(1):98–106. doi: 10.1111/j.1365-2133.2011.10643.x 21929531

[23] Borders CW Courtney A Ronen K Pilar Laborde-Lahoz M Guidry TV Hwang SA. Requisite role for complement C5 and the C5a receptor in granulomatous response to mycobacterial glycolipid trehalose 6,6'-dimycolate. Scand J Immunol (2005) 62(2):123–30. doi: 10.1111/j.1365-3083.2005.01643.x 16101818

[24] Swaisgood CM Ruple LM Culver DA. Complement anaphylatoxins C3a and C5a increase in the bal of mice at the peak of lung granuloma formation using the mycobacterial antigen soda,. Am J Respir Crit Care Med (2011) 183:A4520.

