# Supplementary material for: Complement activation in Hidradenitis suppurativa: Covert low-grade inflammation or innocent bystander?
Source: Front Immunol. 2022 Sep 21;13:953674. doi: 10.3389/fimmu.2022.953674 (PMC9535337; doi:10.3389/fimmu.2022.953674)
Supplement: Supplementary file 1 [file Table_1.docx]

**Supplemental Table 1.** **Antibodies and dilutions used in the study**

| **Antibody** | **Host and targets species** | **Supplier** | **Catalog number** | **Concentration/dilution** |
| --- | --- | --- | --- | --- |
| **C1q** | Polyclonal goat anti-human, FITC labeled | Roche | 760-2688 | 66.6 µg/ml |
| **C4d** | Polyclonal rabbit anti-human C4d | Biomedica | BI-RC4D | 1:600 |
| **Properdin** | Polyclonal rabbit anti-human properdin | Kindly provided by prof. M.R. Daha, Leiden. | n.a. | 1:400 |
| **C3c** | Polyclonal rabbit anti-human, FITC labeled | Dako | F0201 | 1:200 |
| **C5b-9** | Polyclonal rabbit anti-human C5b-9 | Abcam | ab55811 | 1:800 |
| **C5aR1** | Monoclonal mouse anti-human C5aR1 (S5/1) | Hycult | HM2094 | 1:400 |
